# pMHChat, characterizing the interactions between major histocompatibility complex class II molecules and peptides with large language models and deep hypergraph learning

**DOI:** 10.1093/bib/bbaf321

**Published:** 2025-07-07

**Authors:** Jiani Ma, Zhikang Wang, Cen Tong, Qi Yang, Lin Zhang, Hui Liu

**Affiliations:** School of Information and Control Engineering, China University of Mining and Technology, No. 1 Daxue Road, Tongshan District, Xuzhou, Jiangsu 221116, China; Department of Biochemistry and Molecular Biology, Monash Biomedicine Discovery Institute, Monash University, Wellington Rd, Clayton, Melbourne, VIC 3800, Australia; Western Crop Genetics Alliance, College of Science, Health, Engineering and Education, Murdoch University, South St, Murdoch, Perth, WA 6150, Australia; School of Information and Control Engineering, China University of Mining and Technology, No. 1 Daxue Road, Tongshan District, Xuzhou, Jiangsu 221116, China; School of Information and Control Engineering, China University of Mining and Technology, No. 1 Daxue Road, Tongshan District, Xuzhou, Jiangsu 221116, China; School of Information and Control Engineering, China University of Mining and Technology, No. 1 Daxue Road, Tongshan District, Xuzhou, Jiangsu 221116, China

**Keywords:** MHC class II-peptide binding, large language model, hypergraph convolutional network, residue contact profiling

## Abstract

Characterizing the binding interactions between major histocompatibility complex (MHC) class II molecules and peptides is crucial for studying the immune system, offering potential applications for neoantigen design, vaccine development, and personalized immunotherapy. Motivated by this profound meaning, we developed a model that integrates large language models (LLMs) and deep hypergraph learning for predicting MHC class II–peptide binding reactivity, affinity, and residue contact profiling. pMHChat takes MHC pseudo-sequences and peptide sequences as inputs and processes them through four stages: LLMs fine-tune stage, feature encoding and map fusion stage, task-specific prediction stage, and downstream analysis stage. pMHChat distinguishes itself in capturing contextually relevant and high-order spatial interactions of the peptide–MHC (pMHC) complex. Specifically, in a five-fold cross-validation experiment, pMHChat achieves superior performance, with a mean area under the receiver operating characteristic curve of 0.8744 and an area under the precision–recall curve of 0.8390 in the binding reactivity task, as well as a mean Pearson correlation coefficient of 0.7311 in the binding affinity prediction task. Furthermore, pMHChat also demonstrates the best performance in both the leave-one-molecule-out setting and independent evaluation. Notably, pMHChat can provide residue contact profiling, showing its potential application in recognizing critical binding patterns of the pMHC complex. Our findings highlight pMHChat’s capacity to advance both predictive accuracy and detailed insights into the MHC–peptide binding process. We anticipate that pMHChat will serve as a powerful tool for elucidating MHC–peptide interactions, with promising applications in immunological research and therapeutic development.

## Introduction

Major histocompatibility complex (MHC) class II molecules play a pivotal role in antigen presentation. They function by binding processed extracellular peptides and displaying them on the surface of antigen-presenting cells (APCs) [1–3]. The polymorphic nature of MHC class II molecules defines their binding specificity, enabling them to present a wide variety of peptides to T cells [4]. The resulting peptide–MHC class II (pMHC) complexes are then precisely recognized by the T cell receptor (TCR) on CD4+ T cells through epitope-specific recognition, triggering a series of adaptive immune responses [5]. The precise prediction of MHC class II–peptide binding and the elucidation of their interactions are crucial for understanding T cell activation, immune response efficacy, and pathogen recognition, with far-reaching implications for vaccine development, optimization of immunotherapies, and personalized cancer treatment [6–9].

Recent advancements in sequencing techniques have significantly facilitated the investigation in terms of MHC–peptide binding affinity and reactivity. These technologies, e.g. mass spectrometry (MS)-based immunopeptidomics [10] and MHC-associated peptide proteomics (MAPPS) [11], enable explicit identification and characterization of peptides that are naturally presented on MHC molecules. Nevertheless, despite the large volume of characterized MHC molecule data, only a small subset of them have been experimentally validated for their binding specificities.

Prompted by the extensive polymorphism of MHC molecules and the abundance of available data for further exploration, there is an urgent need to develop *in silico* methods to predict MHC–peptide binding affinity and reactivity, as well as for elucidating the underlying binding mechanisms. Numerous computational methods, particularly those based on deep learning frameworks, offer promising solutions to the challenges associated with MHC–peptide binding specificity. Existing methods can be categorized into two types: (i) allele-specific models, including NetMHC [12], SMM-align [13], and RTA [14] and (ii) pan-specific models, such as NetMHCpan [15, 16], PUFFIN [17], MHCflurry [18, 19], NetMHCIIPan [20], DeepMHCII [21], STMHCpan [22], and ConvNeXt-MHC [23]. However, most of these models are designed for MHC class I molecules. MHC class I molecules are expressed ubiquitously on all nucleated cells, resulting in a substantial amount of data that can be leveraged for model training and validation. Their binding grooves of MHC class I are closed at both ends, accommodating peptides typically around 8–10 amino acids, with 9 being the most common length [24], which enormously simplifies the interaction modelling. In contrast, MHC class II molecules only expressed on APCs, resulting in limited data that poses challenges for effective model training. Additionally, the binding grooves of MHC class II molecules are open at both ends, allowing them to bind longer peptides, generally ranging from 13 to 25 amino acids, with a binding core of 9 or more amino acids [25]. This structural characteristic introduces greater diversity in binding specificities, adding additional complexity and challenge to predictive modelling. Collectively, the above factors highlight the complexity of MHC class II studies, resulting in insufficient attention towards this research topic.

Building upon the NetMHCpan series [15, 16], NetMHCIIPan [20] is the first deep learning model specifically designed to predict MHC class II–peptide binding. NetMHCIIPan introduces pseudo-sequences derived from multiple sequence alignments (MSAs) to replace the full-length, redundant amino acid sequences of MHC molecules, thereby focusing on the highly variable and polymorphic regions. Furthermore, it introduces a gold-standard dataset and developed a robust experimental framework, laying the foundation for subsequent advancements in this research community. However, NetMHCIIPan is based on basic artificial neural networks, which are too simplistic to capture semantically enriched and structurally grounded representations, let alone detailed residue-level binding information. This limitation significantly narrows the scope of its experimental design. Additionally, the model lacks sufficient interpretability from an immunological perspective, potentially reducing transparency and hindering a deeper understanding of the binding process.

Thanks to the development of artificial intelligence, more advanced models, e.g. DeepMHCII [21] and MHCAttnNet [26], have emerged with significantly improved prediction performance. Specifically, DeepMHCII utilizes a binding interaction convolutional layer to effectively learn the representations of interactions between two molecules. Fully connected layers and a max-pooling layer are sequentially leveraged to analyse these interactions and ultimately predict the final binding affinity. MHCAttnNet leverages skip-gram models to generate 1-g embeddings for peptides and 3-g embeddings for MHC molecules, then utilizing bidirectional long short-term memory (BiLSTM) layers to capture semantic features. Additionally, MHCAttnNet incorporates attention mechanisms that identify specific trigrams and corresponding amino acids in MHC molecules that contribute most to the model’s predictions, thereby offering valuable insights into the binding process.

In addition to algorithms specifically tailored for 9-mer peptides to MHC class I molecules binding prediction, several scalable models also serve as references for our research. For instance, Ye *et al*. [22] developed STMHCpan, a model that uses a Star-Transformer architecture to predict MHC class I molecule binding with peptides, particularly for tumour neoantigen presentation. STMHCpan effectively captures both local relationships and long-range dependencies, and its attention mechanism enables accurate identification of anchor residues within the peptide binding core.

Despite significant advancements in MHC-binding prediction, several challenges remain in accurately modelling MHC class II molecule–peptide interactions:

(i) Inadequate modelling of MHC pseudo-sequences: current methods applied natural language processing-based modules like convolutional neural network, BiLSTM, and Transformer architectures to process MSA sequences. However, these modules are not specifically designed or pre-trained for MSA. This may prevent them from effectively capturing the patterns essential to MHC structure and function.

(ii) Insufficient investigation of residue-specific and structure-aware features of peptides: due to the scarcity of experimentally validated pMHC complex data, existing models rely heavily on sequence-based feature extraction. However, MHC–peptide binding interactions occur at the residue level, particularly within the binding groove and binding core. Methods limited to sequence-level feature engineering are short of extracting granularity features for interaction modelling. Furthermore, while protein sequences inherently encode implicit structural information, sequence-based models fall short of it, missing crucial insights that could improve prediction accuracy and deepen understanding of MHC–peptide binding mechanisms.

(iii) Limited insight into residue-level interactions: due to the absence of annotations for residue-residue interactions, existing methods are significantly confined to predicting binding affinity or reactivity. Although methods, e.g. MHCAttnNet and STMHCpan, have attempted to explore binding mechanisms—using attention mechanisms to identify key residues within peptide binding cores, they cannot map the detailed residue-residue contact landscape between the binding groove and binding core, leaving crucial interaction details obscured.

Therefore, we proposed a biologically informed deep learning framework, termed pMHChat, to comprehensively characterize the interactions between MHC class II molecules and peptides. With the employment of the fine-tuned LLMs and HyperConv module, pMHChat demonstrates superior performance in five-fold cross-validation, independent testing, and leave-one-molecule-out (LOMO) experimental settings. Feature space visualization reveals pMHChat’s capability to discern high-order and intricate patterns within the data. Ablation studies of ESM-MSA-1b highlight the advantages of its contextually relevant and MSA-optimized architecture. Furthermore, compared to pairwise graph neural networks (GNNs), HyperConv effectively bridges the sequence-to-structure gap by flexibly capturing spatial interactions and learning high-order structural information. Its effectiveness has been validated in various tasks, including peptide structure prediction [27], drug–disease association prediction [28], and miRNA–disease prediction [29]. We also applied pMHChat to true pMHC complexes, and post-analysis results indicate its potential to identify peptide binding cores. Ultimately, downstream analysis demonstrates the application potential of pMHChat in illuminating the binding surface (i.e. hydrogen bonds, hydrophobic interactions) via providing residue contact profiling of pMHC complexes.

## Materials and methods

### Datasets

#### BD2016

BD2016 [30] (https://services.healthtech.dtu.dk/suppl/immunology/NetMHCIIpan-3.2/) is the golden standard dataset initially developed for training NetMHCIIpan-3.2. It includes 134 281 MHC–peptide binding affinity records, spanning 36 HLA-DR, 27 HLA-DQ, 9 HLA-DP, and 8 H-2 molecules. The experimentally derived IC50 values were normalized to range [0,1] using log50k transform [i.e. 1-log50k(affnM)]. For the binding reactivity task, MHC–peptide pairs with binding affinities above 0.426 are classified as binding. Notably, BD2016 offers a redundancy-reduced, five-fold cross-validation version, where peptides sharing similar motifs are assigned to the same fold. The redundancy-reduced partitioning strategy challenges the binding prediction models in mitigating the potential data shifting and enhancing their generalization.

#### BC2015

The BC2015 dataset comprises 51 pMHC complexes with crystal structures from the Protein Data Bank (PDB), specifically detailing the binding core regions.

#### BD2024

We downloaded the latest MHC class II–peptide binding dataset (2024-02-09) from http://tools.iedb.org/auto_bench/mhcii/weekly/ to construct an independent test dataset, including both affinity and reactivity datasets. MHC class II–peptide pairs present in the BD2016 cohort were filtered out to ensure complete independence. As the results, the independent affinity test dataset comprises 1893 MHC class II–peptide pairs, and the independent reactivity test dataset comprises 2885 pairs.

### pMHChat workflow

**Figure 1:**
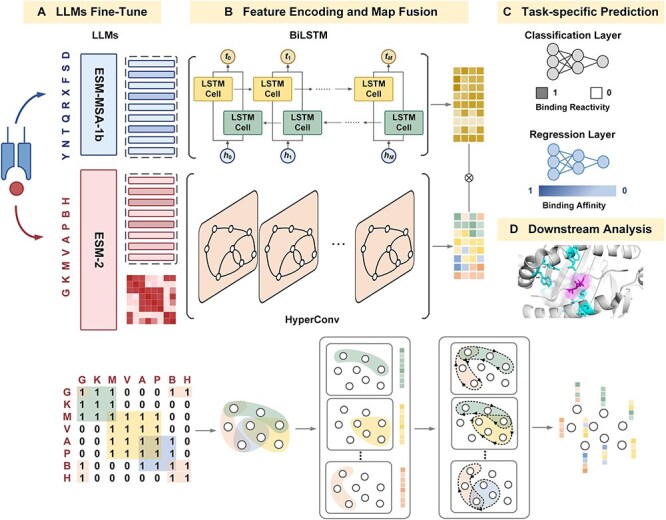
The workflow of pMHChat for task-specific prediction and biologically informed analysis of MHC class II-peptide binding. pMHChat takes MHC pseudo-sequences and peptide sequences as inputs and processes them through four stages: LLMs fine-tune stage, feature encoding and map fusion stage, task-specific prediction stage, and downstream analysis stage. (A) The ESM-MSA-1b and ESM-2 were fine-tuned by solving binding reactivity prediction task, and generated MHC position embeddings, peptide residue embeddings and a residue contact map of the peptide for downstream modelling. (B) pMHChat used BiLSTM to further explore the long-range dependencies amongst different MHC positions. By employing the residue-level features as nodes and local fragments defined from the contact map as hyperedges, HyperConv propagates and updates the residue-level features in accordance with the spatial knowledge. Afterwards, we fused the updated MHC position embeddings and peptide residue embeddings to represent the complex. (C) Regression layer and classification layer (each comprising three MLP layers) were utilized to predict the binding affinity and binding reactivity, respectively. (D) pMHChat can provide the residue contact profiling of the binding surface with downstream analysis.

Here, we developed pMHChat for task-specific prediction and biologically informed analysis of MHC class II–peptide binding (Fig. 1). Taking MHC class II pseudo-sequences and peptide sequences as inputs, pMHChat first undergoes an independent fine-tuning stage, where large language models (LLMs), including ESM-MSA-1b and ESM-2, are fine-tuned to produce contextual and binding-aware residue embeddings for the binding reactivity prediction task. As a byproduct, ESM-2 can generate a predicted residue contact map for the peptide (Fig. 1A). A BiLSTM module was further deployed on MHC residue embeddings to learn long-term bidirectional dependencies amongst MHC residues. Taking residue-level information as node features and local fragments that are defined from the predicted peptide contact map as hyperedges, HyperConv facilitates the propagation of high-order information amongst structurally proximal amino acids, thereby yielding peptide representations that are both semantically rich and structurally grounded. To simulate the interaction process, we fused MHC position embeddings with peptide residue embeddings via an inner product operation. The fusion intends to capture granular interaction patterns between MHC and peptide, thereby generating a binding-aware feature representation of the potential pMHC complex (Fig. 1B). Regression and classification layers were separately applied on it to perform task-specific predictions (Fig. 1C). Notably, for cases predicted as pMHC complexes, pMHChat offers a granular residue contact profiling between the MHC binding groove, shedding light on the interactions within the binding interface (Fig. 1D).

The key stages of pMHChat are described in the following:

#### L‌LMs fine-tuning stage

Here, we fine-tuned ESM-MSA-1b [31] to generate position embeddings for MHC class II molecules, ESM-2 [32] to generate residue embeddings and residue contact map for peptide. Specifically, ESM-MSA-1b is an LLM specialized for MSAs. It employs interleaved row and column attention to capture conserved patterns in MSAs, producing embeddings that reflect residue co-evolution associations. ESM-2, in turn, is a general-purpose protein language model trained on protein sequences. It is widely used to extract structural, functional, and evolutionary features, supporting tasks such as residue embedding and contact map construction. To acquire binding-aware information for the downstream tasks, we fine-tuned the LLMs by unfreezing their last layers and introducing an additional MLP layer on BD2016 dataset, with the objective of binding reactivity prediction. Given a set of training data {(*s*, *p*, *y*)|*s*∈S, *p*∈P, *y*∈Y}, the loss function in the fine-tuning stage is formulated as follows:


(1) \begin{equation*} {\mathrm{L}}_{\mathrm{fine}-\mathrm{tune}}\left(\mathrm{S},\mathrm{P},\mathrm{Y};\Theta \right)={\mathrm{E}}_{\left(s,p,y\right)\epsilon \left(\mathrm{S},\mathrm{P},\mathrm{Y}\right)}\left\Vert \mathrm{F}\left(\mathrm{s},\mathrm{p};\Theta \right)\right.-{\left.y\right\Vert}_2^2 \end{equation*}

where S, P, and Y represent the sets of MHC pseudo-sequences, peptide sequences, and binding labels, respectively, *s =* (*s*_1_*, s*_2_*, …, s_M_*) is the MHC pseudo-sequence with *M* positions, and *p =* (*p*_1_*, p*_2_*, …, p_N_*) is the peptide sequence with *N* residues. F(.) denotes the fine-tuned model, Θ = {Θ*_ESM-MSA-1b_*, Θ*_ESM-2_*, Θ*_MLP_*} is the model parameter, where Θ*_ESM-MSA-1b_* denotes the adjustable parameter set in ESM-MSA-1b, Θ*_ESM-2_* denotes the one in ESM-2, and Θ*_MLP_* denotes the parameter set in the followed adjustable MLP layers. By pre-training pMHChat with the aforementioned loss function, it can generate contextual informed and binding-aware position embedding ***H****_mhc_*$\in{\mathbb{R}}^{M\times{D}_{mhc}}$ for MHC, residue embedding ***H****_p_*$\in{\mathbb{R}}^{M\times{D}_p}$ and predicted residue contact map ***A***$\in{\mathbb{R}}^{N\times N}$ for peptide. Additionally, the model weights from this stage are available at https://zenodo.org/records/15057065.

#### Feature encoding and map fusion stage

Here, we extend our approach with two feature encoders: BiLSTM and HyperConv [33]. For the MHC class II molecule, BiLSTM is applied on position-level embedding ***H****_mhc_* to enhance the feature representation by exploring global sequential dependencies, resulting in ***X****_mhc_*.

For peptides, beyond simple local interactions between adjacent residues at the sequence level, they also engage in spatial interactions, such as hydrogen bonds, hydrophobic interactions, and disulphide bridges, where residues that are distant in the primary sequence are close to each other in 3D space. To explicitly address the sequence-to-structure gap, pMHChat leverages a hypergraph for high-order structural modelling. It employs a HyperConv module for fusing residue embeddings ***H****_p_* with contact map ***A***, bridging the gap between 1D sequence data and high-order hypergraph representations. Contact map ***A*** records the structural distance between all residues. For hypergraph construction, we initially cast the contact map ***A*** into a binary incidence matrix ***C*** using a predefined threshold γ. The threshold γ is set based on a predefined ratio, selecting the top percentage of significant contacts of each contact map for hypergraph construction whilst filtering out the rest. In our experiments, we used a 50% ratio to retain the most relevant connections. For any two residues *p_i_*, *p_j_*, if | *p_i_* − *p_j_* | < γ, that is ***C****_ij_* = 1(*i* = 1,2, …, *N*) and 0 vice versa. Derived from ***C***, we defined the hypergraph G = (V, ε) to model the spatial proximity relationships of peptide residues, where V = {*v*1, *v*2, …, *v_N_*} and ε = {*e_1_*, *e*_2_, …, *e_K_*} (with *K* < *N)* denote the hypernodes and hyperedges, respectively. To prevent the extreme case where all hypernodes are only connected in self-loops, we require that there exists at least one hyperedge *e_i_* such that |*e_i_*| ≥2 for *i* = 1, …, *K*, where |*e_i_*| denotes the number of hypernodes in hyperedge *e_i_*. In practical, we defined R*_i_* = {*p_n_*|***C****_in_* = 1}as the set of interactive residues with respect to *p_i_*. This process is iteratively applied across all rows ***C***(*i*,:), *i* = 1,…,*N*, resulting in sets R_1_, R_2_,…,R*_N_*. If ∃|*e_i_*| ≥2, we filtered out duplicates and achieved the set of hyperedges ε = {*e_1_*, *e*_2_, …, *e_K_*} (with *K* < *N)*. Otherwise, the corresponding binding pair (*s*, *p*) can be discarded. Based on the defined hypergraph *G* = (V, ε), we further calculated the hyperedge index matrix $I{\mathrm\epsilon} {\left\{0,1\right\}}^{N\times K}$ which encodes the mapping from nodes to hyperedges.

Taking node embedding ***H****_p_* and hyperedge index matrix ***I*** as input, pMHChat further deploys HyperConv network on *G* = (V, ε), to propagate message beyond pairwise residues in a degree-free manner, exploiting the high-order structure information and unveiling the intricate spatial interactions of residues. Embracing with ‘node-edge-node’ rules, message aggregation on the hypergraph *G* involves two stages: hyperedge aggregation and hypernode aggregation. In the hyperedge aggregation stage, the hypernode in the same hyperedge are aggregated to update the hyperedge embeddings. The calculation process of hyperedge embeddings at l-th layer ${\boldsymbol{M}}_E^{(l)}$ can be formulated as follows:


(2) \begin{equation*} {\boldsymbol{M}}_E^{(l)}={\boldsymbol{I}}^T{\boldsymbol{X}}_p^{\left(l-1\right)}\Phi \end{equation*}

where ${\boldsymbol{X}}_p^{\left(l-1\right)}$ is the residue embeddings that was achieved at the (*l*-1)-th layer, and ${X}_p^{(0)}={\boldsymbol{H}}_p$, $\Phi \in{\mathbb{R}}^{D_p\times{D}_p}$ is the learnable filter matrix for feature projection. In the hypernode aggregation phase, the node feature is updated by aggregating their related hyperedge features:


(3) \begin{equation*} {\boldsymbol{X}}_p^{(l)}={\boldsymbol{IM}}_E^{(l)} \end{equation*}

During the aggregation process, graph normalization is necessary for mitigating the impact of degree variation and smoothing node features. Finally, the message-passing operation is defined as:


(4) \begin{equation*} {\boldsymbol{X}}_p^{(l)}=\sigma \left({\boldsymbol{D}}_v^{-\frac{1}{2}}{\boldsymbol{I}\boldsymbol{D}}_e^{-1}{\boldsymbol{I}}^T{\boldsymbol{D}}_v^{-\frac{1}{2}}{\boldsymbol{X}}_p^{\left(l-1\right)}\Phi \right) \end{equation*}

where ***D****_v_* is the node degree matrix, ***D****_e_* is the hyperedge degree matrix, and σ is the activation function.

Ultimately, max pooling was performed on ***X****_mhc_* and ***X****_p_* along their first dimension for feature fusion, then we operated inner product to obtain the fine-grained interaction feature embedding ***H****_binding_*  $\in{\mathbb{R}}^{M\times N}$ with the focus on the binding surface.

#### Task-specific prediction stage

To systematically unravel the binding landscape between MHC and peptide sequences, pMHChat is designed with a classification layer and a regression layer to predict binding reactivity and binding affinity. Both layers consist of three MLP layers. Taking the flattened interaction feature embedding ***H****_binding_* as input, they separately output the binding probability and predicted binding affinity, providing a comprehensive understanding of the MHC–peptide interaction dynamics.

## Results

### pMHChat outperforms the state-of-the-art methods in five-fold cross-validation and the independent testing

**Figure 2:**
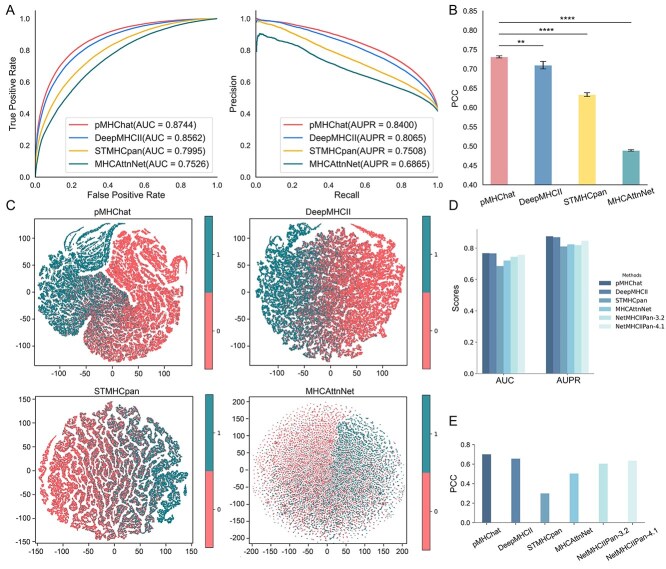
Comparison of the overall performance between pMHChat and other methods on BD2016 (five-fold cross-validation) and BD2024 (independent test). (A) The ROC and PR curves of pMHChat, DeepMHCII, STMHCpan, and MHCAttnNet for the binding reactivity prediction task with five-fold cross-validation. (B) PCC scores of pMHChat, DeepMHCII, STMHCpan and MHCAttnNet for binding affinity prediction task with five-fold cross-validation. (C) Feature embeddings of pMHChat, DeepMHCII, STMHCpan and MHCAttnNet with all test dataset of five-fold cross-validation. (D) AUC and AUPR scores of pMHChat, DeepMHCII, STMHCpan, MHCAttnNet, NetMHCIIPan-3.2, and NetMHCIIPan-4.1 on BD2024 reactivity dataset. (E) PCC scores of pMHChat, DeepMHCII, STMHCpan, MHCAttnNet, NetMHCIIPan-3.2, and NetMHCIIPan-4.1 on the BD2024 affinity dataset.

Binding reactivity prediction is framed as a classification task, which is implemented through the classification layer in the pMHChat framework. To evaluate the capability of pMHChat in this task, we conducted a comparative analysis against three state-of-the-art (SOTA) deep learning models, DeepMHCII, STMHCpan, and MHCAttnNet under five-fold cross-validation setting. All methods were trained and tested using data with identical split settings. The network performance was primarily evaluated using Receiver Operating Characteristic (ROC) curves and Precision–Recall (PR) curves, followed by the Area Under the ROC curve (AUC) and the Area Under the PR curve (AUPR). Detailed description of metrics is listed in Supplementary Material. Then Fig. 2A illustrates the evaluation results of pMHChat and other SOTA methods on the BD2016 five-fold datasets. As shown in Fig. 2A, pMHChat achieves the highest AUC and AUPR, reaching 0.8744 and 0.8380, respectively. pMHChat outperforms DeepMHCII, STMHCpan as well as MHCAttnNet by a large margin of 1.82%, 7.49%, 12.18% in AUC, and 3.35%, 8.92%, 15.35% in AUPR.

Afterwards, we examined the capability of pMHChat in determining the binding affinities between peptides and MHC-II molecules. Binding affinity prediction, as a regression task, is primarily evaluated using the Pearson correlation coefficient (PCC). As shown in Fig. 2B, pMHChat still achieves the highest and relatively stable PCC of 0.7311 ± 0.0028, significantly outperforming the compared SOTA methods. This suggests that the MHC–peptide binding affinities predicted by pMHChat are closest to the experimental results. It is also worth noting that the performance of pMHChat consistently surpasses that of DeepMHCII, followed by STMHCpan and MHCAttnNet across all evaluation metrics and tasks.

To intuitively illustrate the results above, we visualized the distribution of feature embeddings in all folds, underscoring the discriminability of binding pairs and non-binding ones. Figure 2C shows the feature distribution of pMHChat, DeepMHCII, STMHCpan, and MHCAttnNet through t-distributed stochastic neighbour embedding (t-SNE) visualization. pMHChat exhibits a highly complex feature pattern, characterized by the intricate shape of each cluster. Although a small portion of positive samples are slightly overlapped with the negative samples in the feature space of pMHChat, it is still easy to differentiate the positive samples from the negative ones. Additionally, the clusters of positive samples and negative samples in pMHChat’s feature space are tight rather than disperse. In contrast, the feature spaces of DeepMHCII and STMHCpan show a greater mixture of positive and negative samples with feature embeddings displaying a relatively simple pattern. MHCAttnNet performs even worse, as evidenced by a significant overlap between positive and negative samples, along with a simpler and loosely distributed feature space. Thus, the performance of pMHChat can be attributed to its prowess to recognize and harness the distinctive high-order feature patterns of binding pairs and non-binding pairs.

Leveraging the best-performing model in the five-fold cross-validation experiments, we implemented the well-trained pMHChat, DeepMHCII, STMHCpan, and MHCAttnNet on the independent BD2024 test set for generalization evaluation. Additionally, we further compared pMHChat with two benchmark methods, NetMHCIIPan-3.2 and NetMHCIIPan-4.1, in this independent test setting (Fig. 2D and E). Strikingly, pMHChat achieves an AUC of 0.7676 and an AUPR of 0.8752 in predicting MHC–peptide binding reactivity, outperforming all other methods, including DeepMHCII (AUC = 0.7661, AUPR = 0.8692), STMHCpan (AUC = 0.6852, AUPR = 0.8102), MHCAttnNet (AUC = 0.7202, AUPR =0.8237), NetMHCIIPan-3.2 (AUC = 0.7447, AUPR = 0.8188), and NetMHCIIPan-4.1(AUC = 0.7570, AUPR = 0.8469). Additionally, in the binding affinity prediction test, the PCC of pMHChat was 4.39%–40.02% higher than that of the comparison models. The comparison results for both the five-fold cross-validation and independent testing settings are presented in Supplementary Table S1. The computational cost of each method, including the number of parameters and floating point operations per second, is summarized in Supplementary Table S2. Both metrics were calculated during the inference phase using the FlopCount Analysis module from the fvcore.nn Python package. The computations were based on standard input lengths, 34 amino acids for MHC sequences and 15 amino acids for peptide sequences.

### pMHChat shows promising performance in prioritizing peptides for unseen MHC molecules in LOMO

**Figure 3:**
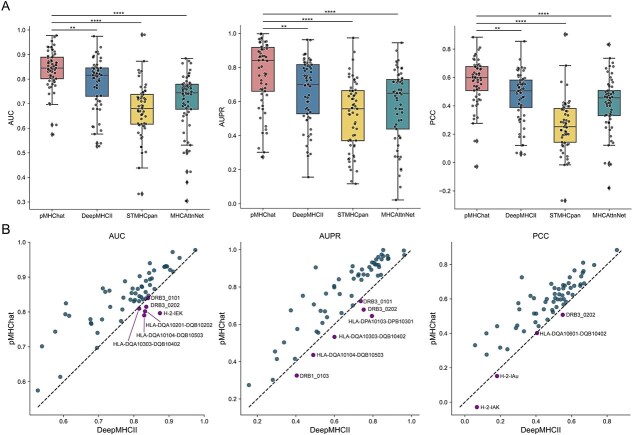
Comparison between pMHChat and other methods on BD2016(LOMO). (A) AUC, AUPR, and PCC distribution of 61 molecules generated by pMHChat, DeepMHCII, STMHCpan, and MHCAttnNet under LOMO. ^**^P-value<.01, ^***^P-value < .001, and ^***^^*^P-value<.0001. (B) Molecule-to-molecule comparison between pMHChat and DeepMHCII under LOMO across metrics.

Following the molecule filtering criteria in DeepMHCII, we selected 61 MHC class II molecules from the 81 molecules in BD2016, ensuring each molecule had more than 40 observations and at least three binding peptides. Instead of using the LOMO setting within a five-fold cross-validation framework as DeepMHCII did, we opted to train the model on 60 molecules at a time, designating the remaining molecules as the test set (Fig. 3). Whilst LOMO under five-fold cross-validation helps mitigate model bias, it may result in an overly optimistic estimate of test performance, as unseen molecule-binding pairs would be distributed across the five test sets. Additionally, it does not accurately reflect real-world scenarios, where the goal is to prioritize peptides for unseen MHC-II molecules. As shown in Fig. 3A, pMHChat consistently demonstrates significant improvements across all metrics under the LOMO setting, achieving the highest mean scores and lowest variance (AUC = 0.8405 ± 0.0770; AUPR = 0.7644 ± 0.1931; PCC = 0.5705 ± 0.1604). The overall ranking of the compared algorithms is highly consistent with previous results, except for slight deviations in the rankings of MHCAttnNet and STMHCpan compared to the five-fold cross-validation and independent test experiments. Although DeepMHCII lags behind pMHChat, it still demonstrates competitive performance across all metrics, significantly outperforming MHCAttnNet and STMHCpan by 7.41%–11.12% in mean AUC, 8.09%–14.02% in mean AUPR, and 4.78%–20.46% in mean PCC. To further explore their capabilities in prioritizing potential peptides for unseen MHC class II molecules, we conducted a molecule-to-molecule comparison between pMHChat and DeepMHCII (Fig. 3B). The analysis revealed that pMHChat outperformed DeepMHCII by achieving higher AUC values for 55 molecules, higher AUPR values for 55 molecules, and superior PCC values for 57 molecules out of a total of 61.

### Fine-tuned LLMs improve the prediction performance with context-aware and evolutionary informed embeddings

To analyse the contribution of fine-tuned LLMs (ESM-MSA-1B and ESM-2), we first compared pMHChat with two modified ones on BD2016 five-fold dataset.

pMHChat (no fine-tuning) denotes a modified pipeline that directly leverages the pre-trained ESM-MSA-1B and ESM-2 models without further fine-tuning. pMHChat (no separate feature encoder) refers to the pipeline that focuses solely on fine-tuning the LLMs, followed by several customized MLP layers, whilst deactivating the functionalities of the BiLSTM and HyperConv components. As shown in Supplementary Table S3, pMHChat (no fine-tuning) exhibits slightly inferior performance to pMHChat on BD2016 dataset across metrics. Notably, even limited to the fine-tuning stage, pMHChat (no separate feature encoder) still surpasses MHCAttnNet and STMHCpan on BD2016 datasets. These results highlight the effectiveness of the fine-tuning process and prompt a deeper exploration of the underlying mechanisms for its effectiveness.

**Figure 4:**
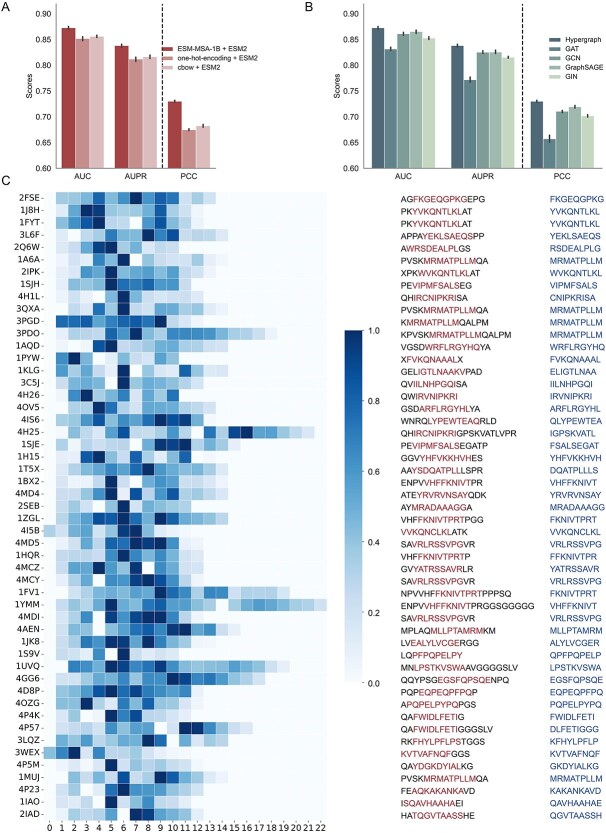
Ablation study and interpretation of LLMs and HyperConv. (A) Performance comparison of pMHChat with ESM-MSA-1b, one-hot-encoding and CBOW encoding on BD2016. (B) Performance comparison of HyperConv and pairwise GNNs on BD2016. (C) Residue importance map, true labelled binding core and predicted binding core of each peptide in BC2015. True binding cores indicated by the left cluster and the predicted binding cores by the right cluster.

ESM-2 provides both contact maps and residue embeddings, which are integrated hierarchically into the HyperConv model and function as a cohesive unit. Given that ESM-2’s contact maps and residue embeddings are already well-integrated with the HyperConv model, we kept ESM-2 unchanged to preserve the structural information in the model. Instead, we selected ESM-MSA-1B as the variable component, sequentially replacing it with alternative representations such as one-hot encoding and Continuous Bag of Words (CBOW) encoding. Figure 4A presents comparative results. As illustrated, the performance remains consistent across metrics. Notably, the pipeline utilizing the ESM-MSA-1B module outperforms the pipeline with the CBOW component, followed by the one using one-hot encoding. These findings align with our expectations and are supported by the following notions. ESM-MSA-1B module is specifically designed to produce context-aware embeddings that encapsulate both contextual and evolutionary information from MSA. Even though CBOW is not the LLM tailored for MSA sequences, it aims at predicting a target residue based on its surroundings, allowing it to learn contextual relationships and generate context-aware residue embeddings. In contrast, one-hot encoding fails to grasp any contextual relationships between residues as it treats each residue as an isolated entity, which explains its poorer performance compared to EMS-MSA-1B and CBOW.

### HyperGCN surpasses pairwise GNNs in capturing high-order residue interactions

Next, we elucidated how HyperConv contributes to the pMHChat. Specifically, we substituted HyperConv with pairwise GNNs, including graph convolutional network (GCN) [34], graph attention network (GAT) [35], graph sample and aggregation network (GraphSAGE) [36], and graph isomorphism network (GIN) [37]. We ran the pipeline with these GNN variants on BD2016 dataset (Fig. 4B). Notably, the performance of pMHChat and the variants with four GNN configurations exhibits consistency across all metrics. HyperConv achieves the best performance with the highest mean AUC, AUPR, and PCC, and the relatively smaller variation. Although GAT and GIN are distinguished for their robust capabilities in graph representation learning, their performance in the MHC–peptide binding prediction task is comparatively limited. This limitation can be attributed to the mismatch between the model’s representational capacity and the length of peptides, where most peptides in BD2016 consist of 8–15 amino acids. Specifically, equipped with an attention mechanism, GAT may introduce excessive complexity for such short peptides, potentially leading to over-smoothing in the representation of graphs. Meanwhile, GIN is specially designed to capture isomorphic graph properties through MLPs for information aggregation, possessing significant power in distinguishing various graph structures. However, when confronted with short peptides, GIN may be overly sensitive to the specificities of the graph structure, potentially overlooking the essential biological patterns behind. In comparison, the message passing and aggregation functions of GCN and GraphSAGE are relatively straightforward. GCN updates node embeddings by averaging the features of all neighbouring nodes. However, using all neighbouring nodes may introduce noise and may cause over-smoothing in small graphs. To mitigate this issue, GraphSAGE first samples a fixed-size set of neighbours of each node, and then updates embeddings by averaging node features. This sampling strategy alleviates the noise and lessens over-smoothing to some extent, thus producing optimistic prediction performance. Thanks to the hyperedge, HyperConv transcends the limitations of pairwise GNNs, offering significant advantages in flexibly learning and capturing intricate high-order residue interactions and complex relational dynamics within peptides. Furthermore, the modelling approach of HyperConv aligns with the reality that any residue can spatially interact with multiple residues through various non-covalent interactions, such as hydrophobic effects and van der Waals forces. Together, these factors ultimately lead to HyperConv’s superior performance in the MHC–peptide binding task.

### Fusion of ESM2 and HyperConv can detect peptide binding cores

To elucidate the underlying rationale for integrating ESM-2 and HyperConv, we pried into the intricate ‘black-box’ formed by their fusion, seeking to unravel the patterns they learned in the context of the MHC–peptide binding prediction task. Leveraging the BC2015 dataset, which contains crystal structures of pMHC complexes with annotated peptide binding cores, we analysed residue-level importance to uncover conserved peptide binding patterns.

To obtain contextual residue contribution, we extracted the attention matrix of ESM-2, which records the correlations amongst all residues. Subsequently, we assessed each residue’s contribution by aggregating its correlation scores with other residues. A higher score indicates a stronger dependency on surroundings, thus highlighting the contribution it provides in MHC–peptide binding process. To gain spatial node importance, GNNExplainer [38] was used to perform post-analysis of the binding process with a node perturbation trick and outputs the spatial residue importance. Taking both contextual and spatial views into consideration, we calculated the ultimate residue importance map by multiplying contextual residue contribution and spatial residue importance (Fig. 4C).

To grasp some hints of peptide binding core from the residue importance map, we employed a sliding window approach to identify regions within the peptide sequences that exhibit the highest cumulative residue importance. Since all the binding cores in BC2015 have a length of nine amino acids, we applied a fixed-length window of length nine to each sequence, traversing with a step size of one. At each step, the window captures a subsequence of nine consecutive residues and the sum of their residue importance scores is calculated. Then, we localized the window that yields the highest cumulative importance score as the most important region that significantly influences the strength and specificity of the MHC–peptide binding. Figure 4C presents the experimentally validated binding cores and predicted important regions for 51 pMHC complexes from the BC2015 dataset. Notably, 35 out of 51 binding cores are fully captured, while 11 other cases exhibit highly overlap with experimentally validated binding cores with a mismatch of only one or two residues shifting. These findings indicate that the fusion of ESM-2 and HyperConv shows great promise in detecting the peptide binding cores, which contributes to the superior performance of pMHChat.

### pMHChat enables residue contact profiling of the binding surface

**Figure 5:**
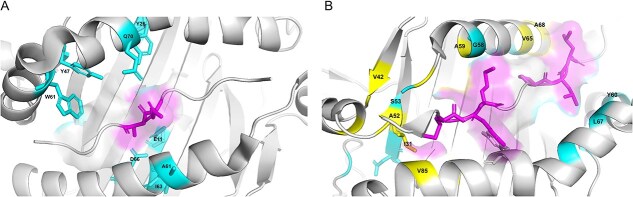
Interaction patterns on the MHC–peptide binding surface (examples). (A) 2Q6W. (B) 3PDO.

The pMHC complex is formed through covalent and non-covalent interactions between residues in the MHC molecule’s binding groove and those in the peptide binding core. These interactions, including hydrogen bonding, disulphide bond, and hydrophobic interactions, stabilize the peptide within the MHC binding groove. To gain more detailed residue contact information between the MHC groove and binding core, we applied the well-trained pMHChat on BC2015, extracted the fine-grained interaction feature embedding matrix ***H****_binding_.* The matrix was then normalized to a range of [0,1], providing a quantitative representation of the relative binding strengths amongst residues. To better interpret the binding patterns, we used PyMOL to examine potential binding interactions in regions exhibiting strong binding affinities. Taking pMHC complexes 2Q6W and 3PDO as examples, Supplementary Fig. S1 displays their residue contact profiling, and Fig. 5 shows the critical interaction patterns found with PyMOL. Supplementary Fig. S1A illustrates that the peptide fragment ‘EA’ exhibits the strongest binding interaction with the ‘QLDYCELFLWMIA’ region of the MHC binding groove. Targeting the ‘EA’ fragment within the binding core on Chain C, we conducted an iterative search for potential hydrogen bonds (within a 3.5 Å range), hydrophobic interactions (within a 3.5 Å range) and disulphide bonds between ‘EA’ and residues on Chains A and B. As shown in Fig. 5A, ‘EA’ forms hydrogen bonds with residues such as Q70, D66, Y26, E11, and Y47, partially aligning with the corresponding regions in the residue contact profiling. Additionally, Supplementary Fig. S1B reveals that the peptide fragments ‘MRM’ and ‘PLL’ are strongly correlated with ‘IADVAAGSA’. Rooted in ‘MRM’ and ‘PLL’, we searched for potential interactions with residues in Chain A and Chain B. Figure 5B shows that ‘MRM’ and ‘PLL’ interact with residues V42, A52, V85, A59, V65, and A68 through hydrophobic interactions and are anchored to S53, G58, L67, and Y60 through hydrogen bonds, interpreting the residue contact profiling in Supplementary Fig. S1B. These findings indicate that the pMHChat has the potential in effectively capturing the crucial binding patterns on the binding surface.

## Discussion

In this study, we proposed a biologically informed deep learning workflow for characterizing MHC class II molecule–peptide binding and providing the residue contact profiling on binding surface. Its performance is notably superior across various testing scenarios, including five-fold cross-validation, independent test setting, and LOMO setting. The promising performance of pMHChat can be attributed to two key architectural strategies. First, the fine-tuned ESM-MSA-1b and ESM-2 are spatially designed to produce context-aware embeddings that encapsulate both contextual and evolutionary information. Secondly, HyperConv captures complex spatial dependencies by constructing hyperedges from ESM-2-derived contact maps. This approach overcomes the limitations of pairwise GNNs and better reflects the reality that any residue can spatially interact with multiple residues through non-covalent interactions. Notably, pMHChat also infers peptide binding cores and residue-level contact maps of the binding surface without explicit training, through the combined use of ESM-2 attention maps and HyperConv-derived node importance. This integration successfully identified nearly 46 out of 51 pMHC complexes in BC2015, underscoring the model’s interpretability and structural awareness.

Despite the strengths, pMHChat still exhibits three principal limitations. First, it is found to be constrained by the limited coverage of MHC molecules, MHC class I may broaden its applicability. Secondly, pMHChat works on predicting peptide–MHC binding rather than identifying epitopes that can be recognized by TCR. Epitope information can offer clues about binding affinity, as certain ones tend to exhibit stronger binding to MHC molecules. Thirdly, the predicted residue contact maps generated by ESM-2 may be biased, potentially affecting the quality of downstream hypergraph construction. This limitation underscores the importance of incorporating more robust structural information to enhance model reliability. Future work could explore utilizing 3D structural data to achieve a more comprehensive and precise representation, thereby improving the overall modelling process and the prediction performance. Moreover, binding core identification is a crucial task. However, due to the design of pMHChat, it was not explicitly trained for this purpose. Future work could focus on optimizing the model to enhance its capability in binding core identification. Finally, the utility of pMHChat is currently limited by its existing application scope. Enhancing its ability to infer neoantigens for tumour-specific immune responses could greatly expand its practical applications, particularly in cancer immunotherapy.

**Key Points**

- pMHChat is a large language models (LLMs)- and hypergraph-based deep learning framework, predicting the binding affinity and reactivity between major histocompatibility complex (MHC) class II molecules and peptides through illuminating the residue contact profiling of the binding surface.
- Fine-tuned LLMs capture evolutionarily conserved, binding-aware patterns, while hypergraph bridges the sequence-to-structure gap by flexibly modelling peptide spatial interactions and leveraging high-order structural information.
- pMHChat excels in five-fold cross-validation and independent testing by capturing complex patterns and accurately distinguishing binding from non-binding pairs. Its strong leave-one-molecule-out performance further underscores its potential for prioritizing peptides for unseen MHC class II molecules.
- pMHChat still has potential in identifying high-resolution residue contact profiling, even in the absence of explicit annotations, offering profound biological insights into MHC class II–peptide interactions.

## Supplementary Material

FigS1_bbaf321

Table_S1_bbaf321

Table_S2_bbaf321

Table_S3_bbaf321

pMHChat-SUPPL_bbaf321

## Data Availability

BD2016 dataset can be accessed at https://services.healthtech.dtu.dk/suppl/immunology/NetMHCIIpan-3.2/. BD2024 and BC2015 can be found at https://github.com/jianiM/pMHChat. Python scripts for pMHChat and its comparative methods are archived in https://github.com/jianiM/pMHChat.
