# Supplementary figures and images for: pMHChat, characterizing the interactions between major histocompatibility complex class II molecules and peptides with large language models and deep hypergraph learning
Source: Brief Bioinform. 2025 Jul 7;26(4):bbaf321. doi: 10.1093/bib/bbaf321 (PMC12229989; doi:10.1093/bib/bbaf321)

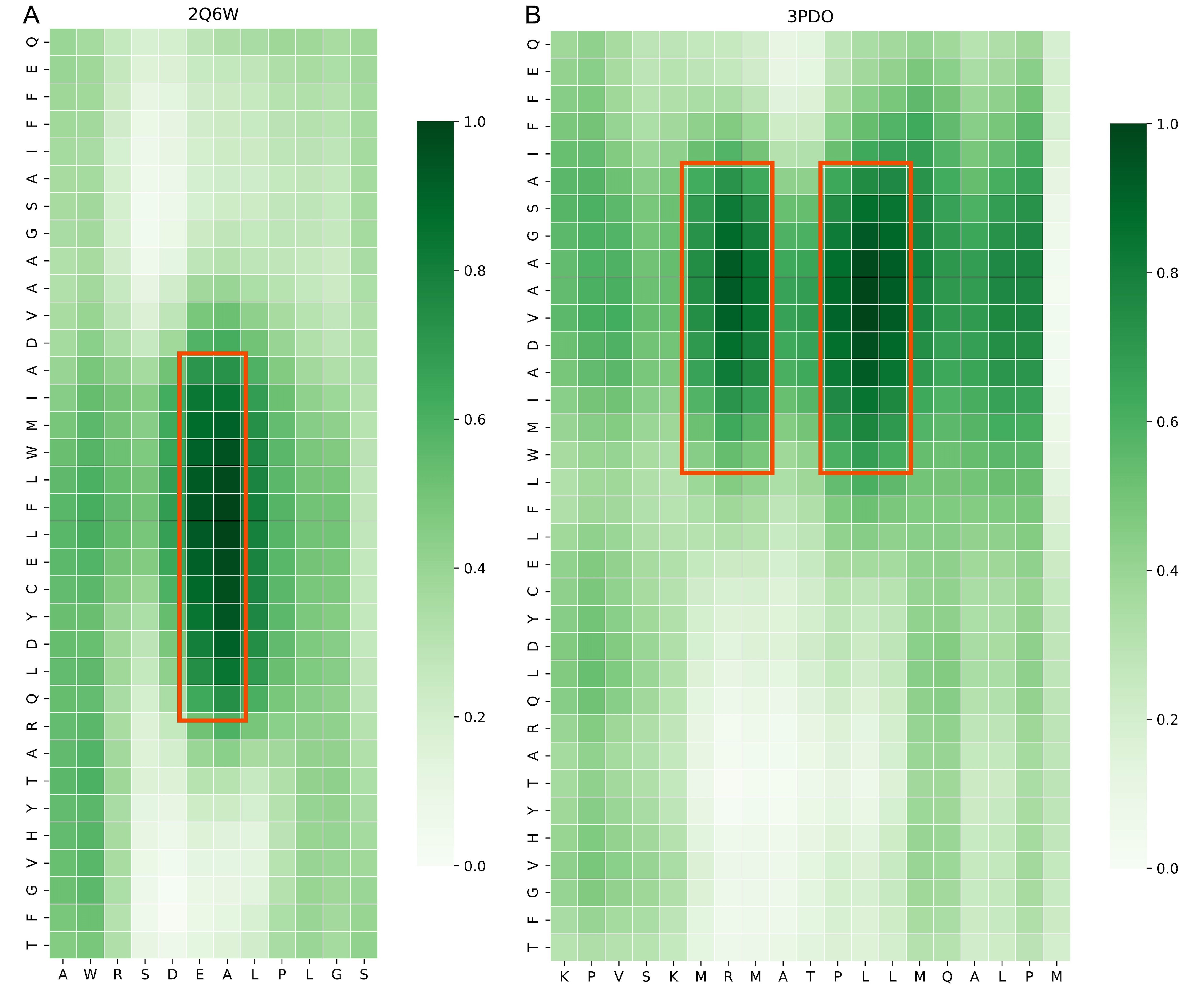

Supplement: FigS1_bbaf321 [file figs1_bbaf321.jpeg]
