# Supplementary material for: pMHChat, characterizing the interactions between major histocompatibility complex class II molecules and peptides with large language models and deep hypergraph learning
Source: Brief Bioinform. 2025 Jul 7;26(4):bbaf321. doi: 10.1093/bib/bbaf321 (PMC12229989; doi:10.1093/bib/bbaf321)
Supplement: Table_S1_bbaf321 [file table_s1_bbaf321.docx]

**Table S1** Comparison performance of pMHChat and other competing methods

| **Method** | **BD2016**  **（5-fold cross-validation）** | | | | **BD2016**  **（LOMO）** | | | **BD2024（binary）** | | **BD2024（IC50）** |
| --- | --- | --- | --- | --- | --- | --- | --- | --- | --- | --- |
|  | AUC | AUPR | PCC | AUC | | AUPR | PCC | AUC | AUPR | PCC |
| **NetMHCIIPan-3.2** | - | - | - | - | | - | - | 0.7447 | 0.8188 | 0.6045 |
| **NetMHCIIPan-4.1** | - | - | - | - | | - | - | 0.7570 | 0.8469 | 0.6344 |
| **DeepMHCII** | 0.8562 | 0.8065 | 0.7096 | 0.7871 | | 0.6600 | 0.4682 | 0.7661 | 0.8692 | 0.6563 |
| **STMHCpan** | 0.7995 | 0.7508 | 0.6335 | 0.6759 | | 0.5197 | 0.2636 | 0.6852 | 0.8102 | 0.3000 |
| **MHCAttnNet** | 0.7526 | 0.6865 | 0.4890 | 0.7130 | | 0.5790 | 0.4205 | 0.7202 | 0.8237 | 0.5036 |
| **pMHChat** | **0.8744** | **0.8400** | **0.7311** | **0.8405** | | **0.7644** | **0.5705** | **0.7676** | **0.8752** | **0.7002** |

“-” indicates that the value does not exist.
