# Supplementary material for: pMHChat, characterizing the interactions between major histocompatibility complex class II molecules and peptides with large language models and deep hypergraph learning
Source: Brief Bioinform. 2025 Jul 7;26(4):bbaf321. doi: 10.1093/bib/bbaf321 (PMC12229989; doi:10.1093/bib/bbaf321)
Supplement: Table_S2_bbaf321 [file table_s2_bbaf321.docx]

**Table S2** Computational cost of pMHChat and other competing methods

| **Method** | **pMHChat** | **DeepMHCII** | **STMHCpan** | **MHCAttnNet** |
| --- | --- | --- | --- | --- |
| **Number of parameters** | 125.37M | 0.53M | **0.26M** | 0.04 M |
| **FLOPs** | 15.73G | 92.2M | **9.21M** | 0.38 M |
