# Supplementary material for: pMHChat, characterizing the interactions between major histocompatibility complex class II molecules and peptides with large language models and deep hypergraph learning
Source: Brief Bioinform. 2025 Jul 7;26(4):bbaf321. doi: 10.1093/bib/bbaf321 (PMC12229989; doi:10.1093/bib/bbaf321)
Supplement: Table_S3_bbaf321 [file table_s3_bbaf321.docx]

**Table S3** Comparative results of two pLM ablation models under 5-fold CV scheme

|  | **Mean AUC** | **Mean AUPR** | **Mean PCC** |
| --- | --- | --- | --- |
| pMHChat(no fine-tune) | 0.8656 | 0.8271 | 0.7011 |
| pMHChat(no separate feature encoders) | 0.8123 | 0.7569 | 0.6371 |
