# Supplementary material for: pMHChat, characterizing the interactions between major histocompatibility complex class II molecules and peptides with large language models and deep hypergraph learning
Source: Brief Bioinform. 2025 Jul 7;26(4):bbaf321. doi: 10.1093/bib/bbaf321 (PMC12229989; doi:10.1093/bib/bbaf321)
Supplement: pMHChat-SUPPL_bbaf321 [file pmhchat-suppl_bbaf321.docx]

### **pMHChat, Characterizing the Interactions Between MHC Class II Molecules and Peptides with LLMs and Deep Hypergraph Learning**

**Supplementary Material**

**Jiani Ma^1^, Zhikang Wang^2^, Cen Tong^3^, Qi Yang^1^, Lin Zhang^1^, Hui Liu^1,*^**

**1** School of Information and Control Engineering, China University of Mining and Technology, Xuzhou, 221116, China

**2** Monash Biomedicine Discovery Institute and Department of Biochemistry and Molecular Biology, Monash University, Melbourne, VIC 3800, Australia

**3.** Western Crop Genetics Alliance, College of Science, Health, Engineering and Education, Murdoch University, Perth, WA, 6150, Australia

*To whom correspondence should be addressed:

hui.liu@cumt.edu.cn

**Metrics used to assess model performance.**

ROC and PR curves were utilized to intuitively evaluate the overall performance of binding reactivity pMHChat and other existing methods. ROC curve shows the trade-off between true positive rate (tpr) and false positive rate (fpr), as the classification threshold varies, which illustrates how well the classifier can distinguish between positive and negative instances for various threshold settings. The PR curve is more informative than AUROC when testing imbalanced data sets, highlighting the balance between precision and recall. Area under the ROC curve (AUC) and area under the PR curve (AUPR), whose values range from 0 to 1, are computed based on the ROC and PR curves, respectively, providing a quantitative measurement of the overall performance of classifiers.

$\text{tpr}=\text{recall}=\frac{\text{TP}}{\text{TP+FN}}$ (1)

$\text{fpr}=\frac{\text{FP}}{\text{FP+TN}}$ (2)

$\text{precision}=\frac{\mathrm{TP}}{\mathrm{TP}+\mathrm{FP}}$ (3)

where TP, FP, TN, and FN denote the numbers of the true positive samples, false positive samples, true negative samples, and false negative samples, respectively.

Pearson correlation coefficient (PCC) was used to evaluate how well the predicted binding affinities correlate with the true binding affinities in the binding affinity prediction task.

$r=\frac{\sum(y_{\text{true}}-\bar{y}_{\text{true}})(y_{\text{pred}}-\bar{y}_{\text{pred}})}{\sqrt{\sum(y_{\text{true}}-\bar{y}_{\text{true}})^{2}\sum(y_{\text{pred}}-\bar{y}_{\text{pred}})^{2}}}$ (4)

where *y*_true_ records the true binding affinities between MHC class II molecules and peptides, while *y*_pred_ records the predicted binding affinities. $\bar{y}_{\text{true}}$ and $\bar{y}_{\text{pred}}$ are the means of the respective values.

**Table Caption:**

**Table S1** Comparison performance of pMHChat and other competing methods

**Table S2** Computational cost of pMHChat and other competing methods

**Table S3** Comparative results of two pLM ablation models under 5-fold CV scheme

**Figure Legend:**

**Fig. S1 Heatmap of residue contact profiling(examples).** The regions marked by the red boxes indicate strong residue interactions among MHC class II molecules and peptides. **A**. 2Q6W **B**. 3PDO.

**Alt Text**: Heatmaps depicting residue contact profiling between MHC class II molecules and peptides. Panel A for 2Q6W, and Panel B for 3PDO.
